# Performance of the phonatory deviation diagram in monitoring voice quality before and after voice exercise in individuals with Parkinson's Disease

**DOI:** 10.1590/2317-1782/20232021224en

**Published:** 2023-07-17

**Authors:** Hellen Vasconcelos Silva Leal de Lima, Leonardo Wanderley Lopes, Hilton Justino da Silva, Ana Cláudia de Carvalho Vieira, Thalita Vitória Silva da Cruz, Adriana de Oliveira Camargo Gomes, Zulina Souza de Lira

**Affiliations:** 1 Programa de Pós-Graduação em Saúde da Comunicação Humana, Centro de Ciências da Saúde, Universidade Federal de Pernambuco - UFPE - Recife (PE), Brasil.; 2 Departamento de Fonoaudiologia, Universidade Federal da Paraíba - UFPB - João Pessoa (PB), Brasil.; 3 Departamento de Fonoaudiologia, Centro de Ciências da Saúde, Universidade Federal de Pernambuco - UFPE - Recife (PE), Brasil.

**Keywords:** Voice, Parkinson Disease, Voice Quality, Acoustics, Speech, Language and Hearing, Sciences

## Abstract

**Purpose:**

To assess the performance of the phonatory deviation diagram and its measurements in monitoring voice quality before and after voice exercise in individuals with Parkinson's Disease.

**Methods:**

Retrospective and documentary study. A sample of 30 subjects was used, 50% male, with a mean age of 62.13 ± 9.05 years. The results of the phonatory deviation diagram were analyzed, in the moments before and after vocal exercise with the pushing technique associated with plosive sounds, considering the area, density, shape and location of the diagram in the quadrants. For comparison purposes, the acoustic parameters of jitter, shimmer, glottal to noise excitation ratio and results of the auditory-perceptual analysis, carried out in previous research, were also considered, in the pre- and post-technical moments.

**Results:**

Despite the fact that there was no difference in the distribution of samples in the diagram, after performing the vocal technique, a change in the displacement of the diagram towards the area of normality was identified in the visual qualitative analysis, and there was an association between the general degree of vocal deviation in the analysis auditory perception and the area of the diagram. There was an improvement in the shimmer values after the vocal technique.

**Conclusion:**

The displacement of the phonatory deviation diagram towards the area of normality corroborated the results in relation to the general degree of dysphonia, evaluated by the auditory-perceptual analysis and the shimmer results, after the vocal technique. Thus, the diagram shows good performance in monitoring voice quality of individuals with Parkinson’s.

## INTRODUCTION

Parkinson’s disease (PD) is a neurodegenerative pathology that affects dopamine production due to the early death of dopaminergic neurons^(1,2)^. PD patients develop symptoms such as tremor at rest, stiffness, slow movements (bradykinesia), and postural changes^(3,4)^. Their speech^(5-7)^ and voices^(8)^ can also be affected, characterized by reduced vocal fold movements and articulatory amplitude, resulting in imprecise speech, at an irregular rate, and with phonatory instability^(9)^.

Voice disorders generally have multidimensional manifestations, which justifies the recommendation for clinicians to include clinical and instrumental assessments^(10)^. Speech-language-hearing clinical assessment involves the auditory-perceptual judgment of the voice quality and the investigation of vocal behavior. Complementarily, the instrumental approach may include acoustic assessment, allowing inferences on the characteristics of the vocal signal and underlying physiological mechanisms^(11)^.

The acoustic analysis takes measures or performs a visual qualitative analysis. Such measures encompass classical ones, such as jitter, shimmer, fundamental frequency, and glottal noise measures. The visual inspection can be made with spectrography or other tools - such as the phonatory deviation diagram (PDD) - that enable a visual analysis of the vocal signal^(12)^.

PDD or hoarseness diagram (as originally proposed)^(12)^ is a multivariate acoustic analysis tool, whose bidimensional chart relates acoustic measures - jitter, shimmer, correlation, and glottal-to-noise excitation ratio (GNE) - obtained from the vocal signal. The diagram provides a visual qualitative analysis, verifying the voice displacement in PDD, also enabling descriptions parameters (area, quadrant, density, and shape)^(13)^. PDD has been used to monitor patients with sequelae of head and neck cancer, vocal fold paralysis, and so on^(14)^.

Monitoring voices with extreme deviations poses a challenge to voice-related research and clinical practice, as highly disturbed voices cannot be analyzed with classical acoustic measures^(14)^. PDD, on the other hand, enables the assessment of more deviated signals and is, therefore, an alternative to assess them^(15)^ - although the main contribution of this tool is the possibility of monitoring patients before and after interventions^(16)^.

Most studies^(12,14,16)^ that use PDD include the voices of patients with behavioral dysphonia, sequelae of head and neck cancer, or neurological laryngeal disorders. Thus, considering the ever-growing number of older people with PD^(17)^, it is important to understand tools such as PDD that can instrumentally help assess improvements in the patient’s voice quality throughout the therapy.

Therefore, this study aimed to verify the performance of PDD and its measures in monitoring voice quality before and after vocal exercises in PD patients.

## METHODS

### Study design

This retrospective study was approved by the Research Ethics Committee of the Department of Health Sciences at the Federal University of Pernambuco, under evaluation report no. 4.322.413. It was based on secondary data, whose database has voice records, auditory-perceptual analysis of the voices, and data on the collection participants regarding the characterization of the sample.

### Sample

The research was developed from a database with samples of 30 PD patients - 15 males and 15 females -, with a mean age of 62.13 ± 9.05 years. Data were collected and analyzed in the voice laboratory of a public university in 2018 and 2019. Since it used the database of another research, approved by the Human Research Ethics Committee under evaluation report no. 3.060.851, this study was exempted from providing an informed consent form. The participant selection criteria were as follows: having the autonomy preserved, having a laryngeal examination with no structural changes, having vocal complaints, and being diagnosed with PD classified in stages I, II, or III, according to the Hoehn & Yahr (HY) scale^(18)^. Individuals with other neurological diseases, motor disability, a history of head and neck surgery, smokers, alcohol consumers, with influenza and/or an allergic reaction, cognitive decline or communication disorders that might prevent them from understanding the intervention instructions, and laryngeal structural changes were excluded from the research.

The voice samples used in this research had been previously recorded and edited in a previous study^(19)^. They were recorded in VoxMetria software, with the sustained 5-second emission of vowel /ε/, in habitual frequency and intensity, before and immediately after a vocal intervention that consisted of pushing with plosive sounds (syllable: pa) - a technique that aims to improve glottal closure^(9)^. The intervention exercises lasted 3 minutes and 2 seconds - 3 minutes of exercises divided into three 1-minute stages and 20 seconds divided into two 10-second pauses between them.

The recordings were likewise edited in VoxMetria, eliminating the first and last seconds of emission, due to their greater irregularity, remaining the middle 3 seconds^(19)^.

### Database analysis

Auditory-perceptual analysis results and sample characterization data were initially collected from the database of the previous research.

Based on the edited vowel /ε/ recordings, this study analyzed PDD and parameters (jitter, shimmer, correlation, and GNE) extracted from VoxMetria. Also, PDD results obtained in this study were compared with auditory-perceptual analysis results, recorded before and after the intervention with the vocal technique.

The auditory-perceptual analysis of the voice samples was performed by speech-language-hearing therapists who specialized in voice with a visual analog scale^(20)^. The interrater agreement index with the kappa test was 0,545, with a p-value =< 0.001. As described in the study whose database was used in this research^(19)^, it considered the assessments of the judge whose intrarater agreement was 0.746, deemed substantial in the kappa test.

### Acoustic analysis

As previously mentioned, PDD was extracted from the vowel /ε/ records edited and recorded in Voxmetria. PDD analyzes noise and perturbation parameters in combination, based on four acoustic measures: jitter, shimmer, correlation, and GNE^(13,15,16)^. Jitter assesses instabilities in standard vocal fold oscillation, quantifying frequency variations cycle by cycle, while shimmer quantifies changes in the phonatory cycle amplitude^(21)^. This research extracted PDD to analyze the distribution of vocal signals per area, quadrant, density, and shape, furnished by the software.

**Figure 1:**
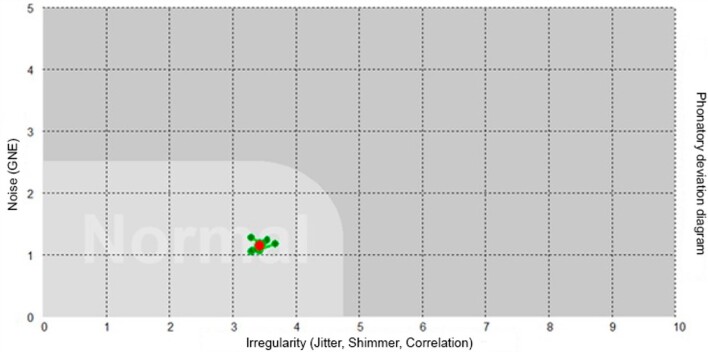
Vowel sample /ε/ from a vocally healthy young adult

Concerning distribution per area, PDD indicates whether the voice sample is within the normal area (Figure 1). Regarding density, the points of vocal signal distribution are classified as concentrated when they occupy a space corresponding to one square (Figure 1). When the voice sample is distributed into more than one PDD square, vocal signals are classified as amplified^(13,16)^. The researcher classified them as either concentrated or amplified by using a common 10-cm ruler.

**Caption:** GNE = glottal-to-noise excitation ratio

**Figure 2:**
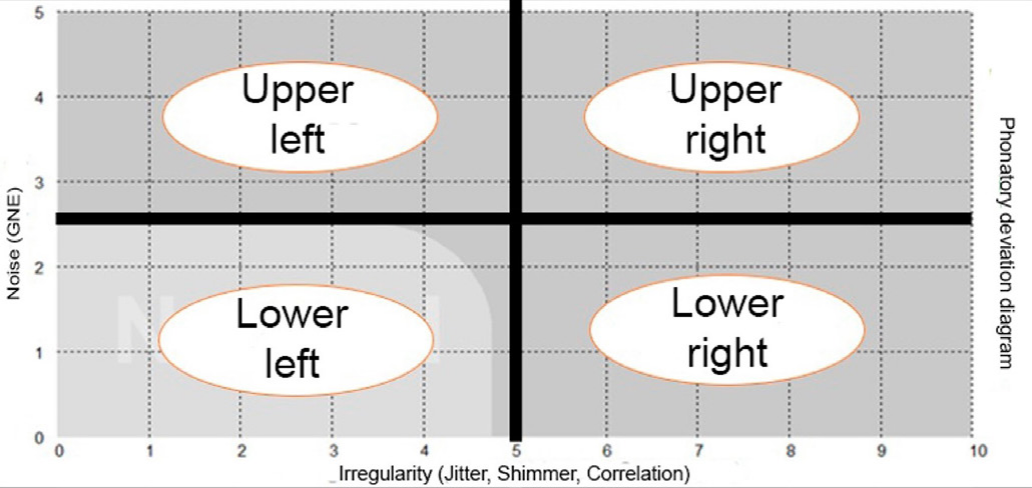
Phonatory deviation diagram divided into four equal quadrants

As for shape, the vocal signal is identified as vertical (when the points cover a longer distance along the vertical line than the horizontal one [y>x]), horizontal (when such distance is greater in the horizontal than the vertical line [x>y]), or circular (when such distances are approximately the same in the vertical and horizontal lines)^(13)^. The shapes were also classified by using the 10-cm ruler. Lastly, PDD was divided into four equal quadrants for distribution into lower-left (1), lower-right (2), upper-right (3), and upper-left (4) (Figure 2)^(13,15,16)^.

Besides analyzing vocal signal distribution per area, quadrant, density, and shape, visual qualitative analysis was performed, verifying PDD displacement in the chart after applying the technique.

### Statistical data analysis

All data were recorded in a 2007 Windows Excel spreadsheet for statistical analysis. Data were organized in a database and analyzed with descriptive statistics.

The Shapiro-Wilk normality test was used to verify whether the sample had a normal distribution, rejecting the hypothesis of normality when p < 0.05. Since the distribution was not normal, the Wilcoxon test was used to compare acoustic parameter values before and after the vocal technique. Homogeneity tests known as chi-square tests were used to verify whether there were differences between frequencies before and after the vocal exercises in each PDD configuration measure.

Association comparison tests (conditional independence) known as Mantel-Haenszel tests were used to assess whether the relationship between auditory-perceptual measures and PDD configuration measures was affected after performing the technique. In all analyses, the level of significance was set at 5%, with a 95% confidence interval.

## RESULTS

The clinical characteristics of PD patients in this research were as follows: regarding the disease stage on the HY scale, 30% were in stage I, 30% were in stage II, and 40% were in stage III. As for clinical type, 53.33% had tremor, 23.33% had stiffness, 6.67% had bradykinesia, and 16.67% has a combination of these.

Table 1 presents the acoustic parameters before and after the vocal technique. Shimmer was the only one that improved after using this vocal technique.

**Table 1:** Acoustic parameters before and after the vocal technique (n: 30)

**Variable**	**Jitter**		**Shimmer**				**Correlation**			**GNE**		
	**Before**	**After**	**p-value**	**Before**	**After**	**p-value**	**Before**	**After**	**p-value**	**Before**	**After**	**p-value**
**Mean** (±SD)	0.68 ± 0.76	0.36 ± 0.50	0.053	9.61 ± 5.59	7.62 ± 4.56	**0.014** *	0.97 ± 0.03	0.98 ± 0.02	0.080	0.68 ± 0.22	0.73 ± 0.21	0.130
**Median**	0.38	0.22		8.70	5.60		0.98	0.99		0.70	0.78	
**(min-max)**	(0.08-3.24)	(0.06-2.79)		(2.63-29.43)	(2.15-19.15)		(0.89-1.00)	(0.88-1.00)		(0.24-0.97)	(0.31-0.98)	
**Quartile 1**	0.175	0.1625		5.430	4.502		0.970	0.980		0.492	0.567	
**Quartile 3**	0.720	0.372		11.475	10.027		0.990	0.990		0.880	0.890	

* Significant values: p ≤ 0.05 - Wilcoxon test

**Caption:** n = sample size; SD = standard deviation; min-max = minimum and maximum values; GNE = glottal-to-noise excitation

PDD area, density, quadrant, and shape configurations before and after the vocal technique are shown in Table 2. The analysis indicated no difference in the distribution of the voice samples after performing the vocal technique. It is important to point out that part of the voices - 14 before and 13 after the technique - was distributed into more than one quadrant. Thus, the total number of voices in the quadrants is greater than that of the sample subjects.

**Table 2:** Area, density, quadrant, and shape configurations of the phonatory deviation diagram before and after the vocal technique (n: 30)

**Configurations**		**Before**		**After**		**p-value** *
		**N**	**%**	**N**	**%**	
**Area**	**Inside**	13	43.3	14	46.7	0.795
	**Outside**	17	56.7	16	53.3	
**Density**	**Concentrated**	17	56.7	15	50.0	0.605
	**Amplified**	13	43.3	15	50.0	
**Quadrant**	**Lower left**	17	56.7	24	80.0	0.381
	**Lower right**	16	53.3	14	46.7	
	**Upper right**	8	26.7	4	13.3	
	**Upper left**	3	10.0	5	16.7	
**Shape**	**Circular**	6	20.0	6	20.0	0.675
	**Horizontal**	16	53.3	13	43.3	
	**Vertical**	8	26.7	11	36.7	

* Chi-square test - Significant values: p ≤ 0.05

**Caption:** n = sample size; % = percentage of the sample

Table 3 shows the relationship between the general degree of vocal deviation and the PDD area before and after the vocal technique. The analysis identified changes in the relationship after the vocal technique - i.e., the number of voices in the neutral and mild degrees increased, while those in the moderate and intense ones decreased. Nonetheless, most voices were still outside the normal area after the technique.

**Table 3:** Relationship between the general degree of vocal deviation and the area of the phonatory deviation diagram before and after the vocal technique (n: 30)

**Area**	**Inside**		**Outside**		**p-value**
**Before**	**N**	**%**	**N**	**%**	**0.000** *
Neutral degree	8	26.7	5	16.7	
Mild degree	5	16.7	3	10.0	
Moderate degree	0	0.0	7	23.3	
Intense degree	0	0.0	2	6.7	
Extreme degree	0	0.0	0	0.0	
Total	13	43.34	17	56.67	
**After**	**N**	**%**	**N**	**%**	
Neutral degree	10	33.3	4	13.3	
Mild degree	4	13.3	6	20.0	
Moderate degree	0	0.0	5	16.7	
Intense degree	0	0.0	1	3.3	
Extreme degree	0	0.0	0	0.0	
Total	14	46.6	16	53.3	

* Significant values: p ≤ 0.05 - Mantel-Haenszel test

**Caption:** n = sample size; % = percentage of the sample

The qualitative analysis verified PDD displacement in the chart to the lower-left quadrant - i.e., closer to the quadrant that represents normality - in 70.58% of the cases after using the technique.

**Figure 3:**
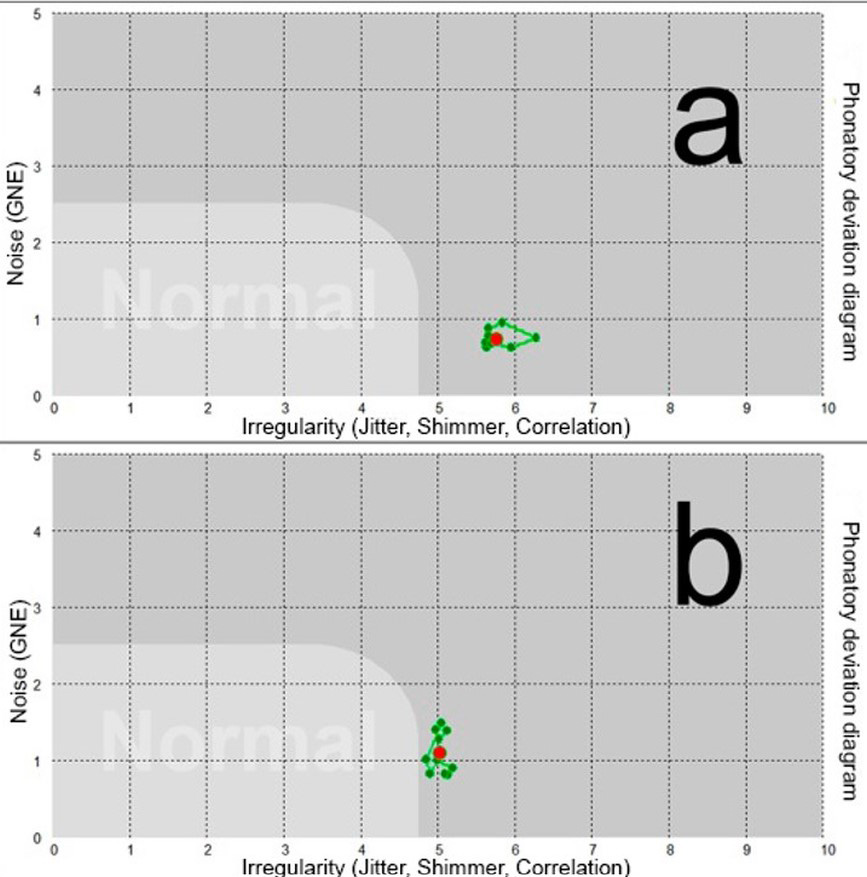
Distributions of vocal samples of a subject of this research, pre and post vocal technique

One of the study participant’s diagrams before and after the technique (Figure 3) was selected to illustrate voice displacement after the vocal technique. It shows that the voice sample is outside the normal area before and after the vocal technique. Qualitatively, it can be observed that PDD displaced to the left in the horizontal axis after the vocal technique.

**Caption:** a = vocal sample before vocal technique; b = vocal sample after vocal technique

## DISCUSSION

PDD is an alternative tool to assess and monitor interventions in extremely deviated voices^(14-16)^. Hence, it can be used in PD patients, as decreases in articulation amplitude and voice quality and intensity are among these individuals’ main communication limitations^(8)^. Therefore, this research aimed to investigate the performance of PDD and its measures to monitor voice quality in PD patients, after the vocal technique of pushing with plosive sounds.

Hence, to base the PDD performance results, the analysis also included individual results of other acoustic parameters and of the auditory-perceptual analysis before and after the vocal technique. The shimmer mean values decreased, which was the only acoustic parameter with a statistically significant difference between means before and after the vocal technique.

Even though these results are above the cutoff (6.5%) for vocally healthy individuals, it can be inferred that improvements in shimmer values after the exercises are related to possible improvements in glottal resistance, as this parameter is sensitive to decreased glottal resistance and is correlated with voice roughness^(22)^. Improved glottal resistance is generally associated with increased vocal intensity - which is one of the main therapeutic goals in PD.

Jitter is a measure strongly correlated with the degree of vocal deviation^(23)^. There was no statistically significant difference in this study between jitter measures before and after the vocal exercise. Nevertheless, this parameter had normal values after the vocal technique, which can be a predictor of overall voice improvement. This result agrees with the auditory-perceptual analysis, as the number of voices in neutral and mild degrees increased and those in the moderate and intense ones decreased after the vocal technique.

It must be highlighted that most participants were older adults, who are expected to have worsened acoustic parameters due to morphological changes in the vocal folds^(24)^. In addition to that, they had PD, in which jitter and shimmer significantly increase^(25)^.

Vocal signal distribution was analyzed per area, quadrant, density, and shape. The comparison before and after the vocal technique revealed no significant difference in the voice sample distribution after the exercises. However, many voices were already in the normal area, which may have influenced their proportion.

It must be also pointed out that the goal of speech-language-hearing therapy in neurodegenerative diseases is to provide communication alternatives, with strategies that improve symptoms as much as possible^(26)^. Thus, speech-language-hearing treatment can improve PD patients’ voices and minimize the impacts of the disease^(27)^, though not necessarily bringing their voices back to a normal standard.

One of the principles of such therapy in PD is to obtain more efficient glottal closure and thus improve the hypophonic characteristic of the emission. This study observed PDD performance by analyzing the effects of a technique that favors glottal closure (pushing with plosive sounds)^(9)^.

Unlike another study that analyzed PDD in different types of dysphonia before and after vocal treatment^(16)^, the present one did not find significant differences in the shape, density, or location in the quadrants between before and after the treatment. On the other hand, despite the absence of such differences in sample distribution, the qualitative analysis, considering proportions, found a trend toward PDD displacement to the left in the horizontal axis after the vocal technique.

This result shows good PDD performance to detect vocal improvement after the exercises, as such displacement brought the diagram to the region nearest to the normal area - which agrees with the acoustic findings of shimmer and overall voice improvements in the auditory-perceptual analysis results.

Furthermore, strained voices are known to tend toward the lower-left quadrant^(13)^. Thus, the vocal exercise increased phonatory effectiveness - i.e., decreased hypophonia. Therefore, PDD can be indicated to monitor vocal hypofunction in PD.

Before the vocal technique, most voices were in the lower-left quadrant, and this number increased after the vocal exercises. Many of the voices analyzed were distributed into more than one quadrant - mostly in the lower-left and lower-right ones, the latter being characteristic of rough voices^(13)^. These findings corroborate the literature, which indicates that roughness is one of the main voice changes in PD^(8,9)^. This characteristic may be related to unstable vocal fold vibration present in this disease.

A study^(15)^ on PDD performance to assess synthesized rough voices also concluded that rougher ones are located in the lower-right quadrant. It also found that the PDD area and quadrant can discriminate rough voices from healthy ones. However, part of the neutral voices in the present study were outside the normal PDD before and after the technique.

Concerning the relationship between the general degree of vocal deviation and the PDD area, it was also noticed that most voices were already in the neutral and mild degrees before the vocal technique, whereas none of the voices was in the extreme degree. After the vocal exercises, the number of voices in the neutral and mild degrees increased and those in the moderate and intense ones decreased. As before the technique, none of the voices had extreme vocal deviation.

The case illustrated in the results shows that after the vocal technique, the voice displaced to the left in the horizontal axis but did not enter the normal area. The original authors of the hoarseness diagram analyzed the distribution of voices of individuals with different voice pathologies^(14)^. A voice highly affected by laryngeal cancer surgery improved after vocal rehabilitation, causing it to significantly displace within PDD. However, after 3 months, it had not yet entered the normal area.

This agrees with the results of the present research. Although most voices in the neutral degree both before and after the vocal technique were inside the normal area proposed by PDD, some of them were outside it. Therefore, it must be considered that these voices had been affected by neurodegenerative disease and that the vocal technique would not necessarily bring them back to a normal standard. Rather, improves vocal efficiency to minimize the impact of the disease on the voice, as previously discussed.

Lastly, the limitations of this study include the sample size (n = 30), which, despite being near that of another study that analyzed PDD in dysphonic voices in general (n = 34)^(16)^, was quite smaller than the sample of research with synthesized voices (n = 871)^(15)^. On the other hand, it is a specific population with the same primary disease, though in different degrees, and the disease limits their possibilities of attending healthcare centers. From this perspective, the number of subjects was quite acceptable. Even so, the study should continue with a larger sample, also addressing different types of dysarthrophonia.

Moreover, there were no differences in the patients’ vocal signal distribution regarding description parameters before and after the intervention. In such cases, speech-language-hearing therapists should perform a visual qualitative analysis, observing the voice displacement in PDD quadrants as they monitor patients in therapy. It is important to reinforce that PDD facilitates the combined analysis of vocal acoustic results and portrays phonatory mechanisms patients are likely to use in speech^(16)^, and the visual qualitative analysis suggested in this study can be useful in such clinical reasoning in these patients’ follow-ups.

Moreover, professionals can also use PDD as an auxiliary tool in therapy, especially to follow up on the progress of patients as a means of visual feedback for them, as they can verify the displacement in the diagram as their voices improve during treatment.

## CONCLUSION

The distribution of patients’ vocal signals regarding description parameters (area, shape, density, and quadrant) was not different before and after the intervention. However, the visual qualitative analysis showed that the vocal signal displaced to the left in the horizontal axis toward a decrease in irregularity.
