# Supplementary material for: Performance of the phonatory deviation diagram in monitoring voice quality before and after voice exercise in individuals with Parkinson's Disease
Source: Codas. 2023 Jul 17;35(4):e20210224. doi: 10.1590/2317-1782/20232021224en (PMC10446749; doi:10.1590/2317-1782/20232021224en)
Supplement: Translation

# Translation: Desempenho do diagrama de desvio fonatório no monitoramento da qualidade vocal pré e pós exercício vocal em indivíduos com Doença de Parkinson

**DOI:** 10.1590/2317-1782/20232021224pt

**Authors:** Hellen Vasconcelos Silva Leal de Lima, Leonardo Wanderley Lopes, Hilton Justino da Silva, Ana Cláudia de Carvalho Vieira, Thalita Vitória Silva da Cruz, Adriana de Oliveira Camargo Gomes, Zulina Souza de Lira

**Affiliations:** 1 Programa de Pós-Graduação em Saúde da Comunicação Humana, Centro de Ciências da Saúde, Universidade Federal de Pernambuco - UFPE - Recife (PE), Brasil.; 2 Departamento de Fonoaudiologia, Universidade Federal da Paraíba - UFPB - João Pessoa (PB), Brasil.; 3 Departamento de Fonoaudiologia, Centro de Ciências da Saúde, Universidade Federal de Pernambuco - UFPE - Recife (PE), Brasil.

**Keywords:** Voz, Doença de Parkinson, Qualidade da Voz, Acústica, Fonoaudiologia

## Abstract

**Objetivo:**

Verificar o desempenho do diagrama de desvio fonatório e de suas medidas no monitoramento da qualidade da voz, pré e pós exercício vocal, em indivíduos com Doença de Parkinson.

**Método:**

Estudo retrospectivo e documental. Utilizou-se uma amostra de 30 sujeitos, sendo 50% do sexo masculino, com média de idade de 62,13 ± 9,05 anos. Foram analisados os resultados do diagrama de desvio fonatório, nos momentos pré e pós exercício vocal com a técnica de empuxo associada aos sons plosivos, considerando-se a área, densidade, forma e localização do diagrama nos quadrantes. Para fins de comparação, os parâmetros acústicos de *jitter, shimmer, glottal to noise excitation ratio* e resultados da análise perceptivo-auditiva, realizada em pesquisa anterior, também foram considerados, nos momentos pré e pós-técnica.

**Resultados:**

A despeito de não ocorrer diferença na distribuição das amostras no diagrama, após realização da técnica vocal, foi identificada, na análise qualitativa visual, mudança no deslocamento do diagrama em direção à área de normalidade e houve associação entre o grau geral do desvio vocal da análise perceptivoauditiva e a área do diagrama. Houve melhora nos valores de *shimmer*, após a técnica vocal.

**Conclusão:**

O deslocamento do diagrama de desvio fonatório em direção à área de normalidade corroborou os resultados em relação ao grau geral de disfonia avaliado pela análise perceptivoauditiva e os resultados de *shimmer*, após a técnica vocal empregada. Desse modo, o diagrama de desvio fonatório apresentou bom desempenho no monitoramento da qualidade vocal de indivíduos com Parkinson.

## INTRODUÇÃO

A Doença de Parkinson (DP) é uma patologia neurodegenerativa que afeta a produção da dopamina devido a morte precoce de neurônios dopaminérgicos^(1,2)^. Pessoas com DP vão desenvolver sintomas como tremor de repouso, rigidez, lentidão do movimento (bradicinesia), e alterações posturais^(3,4)^. Além disso, esses indivíduos podem apresentar alterações na fala^(5-7)^ e voz^(8)^, com características decorrentes da redução dos movimentos das pregas vocais e diminuição da amplitude articulatória, resultando em uma fala imprecisa, com velocidade irregular e instabilidade fonatória^(9)^.

Em geral, o distúrbio de voz apresenta manifestações multidimensionais, o que justifica a recomendação para inclusão da avaliação clínica e instrumental no contexto clínico^(10)^. A avaliação clínica fonoaudiológica é realizada por meio do julgamento perceptivoauditivo da qualidade vocal e da investigação do comportamento vocal. De maneira complementar, a avaliação instrumental pode incluir avaliação acústica, que permite fazer inferências sobre as características do sinal vocal e os mecanismos fisiológicos subjacentes^(11)^.

A análise acústica envolve a extração de medidas ou análise qualitativa visual. A extração de medidas compreende medidas clássicas, como *jitter*, *shimmer*, frequência fundamental, e medidas de ruído glótico. A inspeção visual pode ser realizada por meio de espectrografia ou com ferramentas que possibilitem uma análise visual do sinal vocal, como o diagrama de desvio fonatório (DDF)^(12)^.

O DDF ou *hoarseness diagram* (como proposto originalmente)^(12)^ é uma ferramenta de análise acústica multivariada, correspondendo a um gráfico bidimensional que relaciona as seguintes medidas acústicas obtidas a partir do sinal vocal: *jitter*, *shimmer*, correlação, *glottal to noise excitation ratio* (GNE). A análise com o diagrama é qualitativa visual, verificando o deslocamento da voz dentro do DDF, mas também pode-se usar parâmetros de descrição: área, quadrante, densidade e forma^(13)^. O DDF tem sido utilizado no monitoramento de pacientes com sequelas de câncer de cabeça e pescoço, paralisia de prega vocal, dentre outros^(14)^.

O monitoramento de vozes com desvios extremos é um desafio na clínica e pesquisa na área de voz, pois as medidas acústicas clássicas não permitem a análise de vozes altamente perturbadas^(14)^. Dessa forma, o DDF é uma ferramenta alternativa para avaliação desses sinais, pois permite avaliar sinais mais desviados^(15)^. No entanto, a principal contribuição do DDF está relacionada à possibilidade de monitorar o paciente pré e pós intervenção^(16)^.

A maioria dos estudos^(12,14,16)^ com o DDF incluem vozes de pacientes com disfonias comportamentais, sequelas de câncer de cabeça e pescoço ou alterações neurológicas da laringe. Sendo assim, considerando que há um aumento progressivo da população idosa com DP^(17)^, torna-se importante compreender ferramentas como o DDF, que podem ajudar na avaliação instrumental da melhora da qualidade vocal do paciente ao longo da terapia.

Diante disso, o objetivo do presente estudo é verificar o desempenho do DDF e de suas medidas no monitoramento da qualidade vocal pré e pós exercício vocal em indivíduos com DP.

## MÉTODO

### Desenho do estudo

Trata-se de um estudo retrospectivo, aprovado pelo Comitê de Ética em Pesquisa do Centro de Ciências da Saúde da Universidade Federal de Pernambuco, sob o parecer de n° 4.322.413. É oriundo de dados secundários, cujo banco de dados se constitui em registros vocais, análise perceptivoauditiva das vozes (APA) e dados dos indivíduos participantes da coleta, relativos à caracterização da amostra.

### Amostra

A pesquisa foi desenvolvida a partir de um banco de dados composto pelas amostras de 30 sujeitos, com Doença de Parkinson, sendo 15 do sexo masculino e 15 do sexo feminino, com média de idade de 62,13 ± 9,05 anos. Os dados foram coletados e analisados no laboratório de voz de uma universidade pública durante o período de 2018 a 2019. Por ser desenvolvido a partir do banco de dados de outra pesquisa aprovada no Comitê de Ética em Pesquisa com Seres Humanos, sob o parecer de nº 3.060.851, justifica-se a dispensa do Termo de Consentimento Livre e Esclarecido (TCLE). Foram considerados os seguintes critérios de seleção dos participantes: ter autonomia preservada, possuir exame de laringe sem alterações estruturais, presença de queixa vocal e ter o diagnóstico da DP, classificados nos estágios I, II e III, segundo a escala Hoehn & Yahr (HY)^(18)^. Foram excluídos da pesquisa indivíduos que tivessem outras doenças neurológicas, incapacidade motora, histórico de cirurgia de cabeça e pescoço, fumantes, etilistas e com processos gripais e/ou quadros alérgicos instalados. Possuir declínios cognitivos ou distúrbios de comunicação que impossibilitassem a compreensão das instruções do processo interventivo, e presença de alterações estruturais laríngeas.

As amostras vocais utilizadas nesta pesquisa foram gravadas e editadas previamente, em estudo anterior^(19)^. As gravações foram realizadas utilizando-se o *software* VoxMetria, durante a emissão da vogal /ε/ sustentada, em frequência e intensidade habituais, por cinco segundos. Tais gravações ocorreram antes e imediatamente após a intervenção vocal com a técnica de empuxo associada aos sons plosivos (sílaba: pa). O objetivo dessa técnica foi favorecer a melhora do fechamento glótico^(9)^. O exercício de intervenção teve duração de três minutos e 20 segundos; esse tempo equivale a três minutos de exercícios, sendo esse tempo dividido em três etapas de um minuto, e os 20 segundos de pausas divididas em duas pausas com duração de 10 segundos para cada uma delas.

Os áudios também foram editados no programa VoxMetria, eliminando-se o primeiro e último segundo de emissão, devido à maior irregularidade nesses trechos, permanecendo os três segundos centrais ^(19)^.

### Análise do banco de dados

Inicialmente, foram coletados do banco de dados da pesquisa anterior, os resultados da APA, e as informações relativas à caracterização da amostra.

A partir dos registros editados das gravações da vogal /ε/, o presente estudo contemplou a análise do DDF e dos parâmetros de *jitter*, *shimmer*, correlação e *glottal to noise excitation ratio* (GNE), extraídos do próprio programa VoxMetria. Ademais, foi realizada a comparação entre os resultados obtidos do DDF, no presente estudo, e os resultados da APA, registrados nos momentos pré e pós intervenção com a técnica vocal.

Quanto à APA, vale destacar que foi desempenhada por fonoaudiólogos especialistas na área de voz, considerando a amostra vocal e utilizando a escala analógica visual (EAV)^(20)^. O índice de concordância interexaminadores, pelo teste Kappa, foi de 0,545, com valor de p=<0,001. Conforme descrito no estudo que gerou o banco de dados utilizado na presente pesquisa^(19)^, foram consideradas as avaliações do juiz cuja concordância intraexaminador foi de 0,746, considerado substancial pelo teste Kappa.

### Análise acústica

Como descrito, o DDF foi extraído a partir do registro gravado e editado da vogal /ε/, no *software Voxmetria*. O DDF é utilizado para analisar, de forma combinada, parâmetros de perturbação e de ruído, e é baseado em quatro medidas acústicas: *jitter, shimmer*, correlação e GNE^(13,15,16)^. O *jitter* avalia as instabilidades de oscilação padrão das pregas vocais, quantificando a variação ciclo a ciclo na frequência e o *shimmer* quantifica as mudanças de amplitude do ciclo fonatório^(21)^. O DDF foi extraído, nesta pesquisa, com o objetivo de analisar a distribuição dos sinais vocais de acordo com a área, quadrante, densidade e forma, fornecidos pelo próprio *software.*

Quanto à distribuição em relação à área, o DDF indica se a amostra vocal está dentro ou fora da área de normalidade (Figura 1). Em relação à distribuição vocal relacionada à densidade, os pontos referentes à distribuição dos sinais vocais se classificam como concentrados, quando os pontos estão distribuídos em um espaço que corresponde a um quadrado (Figura 1). Quando a amostra vocal se distribui por mais de um quadrado no DDF, os sinais vocais são classificados como ampliados^(13,16)^. Para classificar os sinais vocais como concentrados ou ampliados, a pesquisadora utilizou uma régua simples de 10 cm.

**Figura 1:** Amostra da vogal /ε/ de um adulto jovem vocalmente saudável

**Legenda:** GNE = *glottal to noise excitation ratio*

Com relação à forma, o sinal vocal apresenta-se como vertical quando a distância dos pontos ao longo da linha vertical é maior que a distância dos pontos na linha horizontal (y>x); e corresponde a horizontal quando a distância entre os pontos na linha horizontal é maior que a distância na linha vertical (x>y); circular quando a distância dos pontos na linha vertical e horizontal é aproximadamente igual^(13)^. Na classificação da forma, também se utilizou a mesma régua de 10 cm. Finalmente, quanto à distribuição vocal de acordo com quadrante, o DDF foi dividido em quatro quadrantes iguais: inferior esquerdo (1), inferior direito (2), superior direito (3) e superior esquerdo (4) (Figura 2)^(13,15,16)^.

**Figura 2:** Diagrama de desvio fonatório dividido em quatro quadrantes iguais

Além da análise da distribuição dos sinais vocais de acordo com a área, quadrante, densidade e forma, foi realizada análise qualitativa visual, verificando-se o deslocamento do DDF no gráfico, após a aplicação da técnica.

### Análise estatística dos dados

Todos os dados foram registrados em planilha no programa Excel/Windows 2007 para análise estatística. Os dados foram organizados em um banco de dados e analisados por meio de estatística descritiva.

Para verificar se a amostra apresentava distribuição normal, foi utilizado o teste de normalidade Shapiro-Wilk, rejeitando-se a hipótese de normalidade quando p<0,05. Por não apresentar distribuição normal, utilizou-se o teste de Wilcoxon na comparação dos valores dos parâmetros acústicos pré e pós-técnica vocal. Para identificar se havia diferença entre as frequências pré e pós exercício vocal, para cada uma das medidas de configurações do DDF, foram realizados testes de homogeneidade, conhecidos como Testes Qui-quadrado.

Com o objetivo de avaliar se a relação entre as medidas perceptivoauditivas e as medidas das configurações do DDF foi afetada após realização da técnica, foram realizados testes de comparação de associação (independência condicional), conhecidos como Testes de Mantel-Haenszel. Em todas as análises, o nível de significância adotado foi de 5%, com intervalo de confiança de 95%.

## RESULTADOS

Sobre as características clínicas dos participantes com Doença de Parkinson desta pesquisa: em relação ao estágio da Doença de Parkinson, de acordo com a escala Hoehn & Yahr, 30% dos indivíduos estão no estágio I, 30% no estágio II e 40% no estágio III. Quanto ao tipo clínico, 53,33% apresentam tremor, 23,33% rigidez, 6,67% apresentou bradicinesia e 16,67% misto.

A Tabela 1 apresenta os parâmetros acústicos, nos momentos pré e pós técnica vocal. *Shimmer* foi o único parâmetro acústico que apresentou melhora, após a técnica vocal empregada.

**Tabela 1:** Parâmetros acústicos, nos momentos pré e pós técnica vocal (n: 30)

**Variável**	***Jitter***		***Shimmer***				**Correlação**			**GNE**		
	**Pré**	**Pós**	**p-valor**	**Pré**	**Pós**	**p-valor**	**Pré**	**Pós**	**p-valor**	**Pré**	**Pós**	**p-valor**
**Média (**±DP)	0,68 ± 0,76	0,36 ± 0,50	0,053	9,61 ± 5,59	7,62 ± 4,56	**0,014** *	0,97 ± 0,03	0,98 ± 0,02	0,080	0,68 ± 0,22	0,73 ± 0,21	0,130
**Mediana**	0,38	0,22		8,70	5,60		0,98	0,99		0,70	0,78	
**(mín-máx)**	(0,08-3,24)	(0,06-2,79)		(2,63-29,43)	(2,15-19,15)		(0,89-1,00)	(0,88-1,00)		(0,24-0,97)	(0,31-0,98)	
**1° Quartil**	0,175	0,1625		5,430	4,502		0,970	0,980		0,492	0,567	
**3° Quartil**	0,720	0,372		11,475	10,027		0,990	0,990		0,880	0,890	

* Valores significativos: p ≤ 0,05 - Teste de Wilcoxon

**Legenda:** n = tamanho da amostra; DP = desvio-padrão; mín-máx = valor mínimo e máximo; GNE = *glottal to noise excitation*

As configurações de área, densidade, quadrante e forma do DDF, nos momentos pré e pós técnica vocal, estão na Tabela 2. De acordo com a análise, não houve diferença na distribuição das amostras vocais após realização da técnica vocal. Vale destacar também que parte das vozes, 14 pré técnica e 13 após a técnica, estavam distribuídas em mais de um quadrante. Desse modo, o total de vozes nos quadrantes ultrapassou o número de sujeitos da amostra.

**Tabela 2:** Configurações de área, densidade, quadrante e forma do diagrama de desvio fonatório, nos momentos pré e pós técnica vocal (n: 30)

**Configurações**		**Pré Técnica Vocal**		**Pós Técnica Vocal**		**p-valor** *
		**N**	**%**	**N**	**%**	
**Área**	**Dentro**	13	43,3	14	46,7	0,795
	**Fora**	17	56,7	16	53,3	
**Densidade**	**Concentrada**	17	56,7	15	50,0	0,605
	**Ampliada**	13	43,3	15	50,0	
**Quadrante**	**Inferior Esquerdo**	17	56,7	24	80,0	0,381
	**Inferior Direito**	16	53,3	14	46,7	
	**Superior Direito**	8	26,7	4	13,3	
	**Superior Esquerdo**	3	10,0	5	16,7	
**Forma**	**Circular**	6	20,0	6	20,0	0,675
	**Horizontal**	16	53,3	13	43,3	
	**Vertical**	8	26,7	11	36,7	

* Valores significativos: p ≤ 0,05 - Teste Qui-quadrado

**Legenda:** n = tamanho da amostra; % = percentual da amostra

Na Tabela 3 está a relação entre o grau geral do desvio vocal e área do DDF, nos momentos pré e pós técnica vocal. Com base na análise realizada, foi identificada mudança na relação após técnica vocal, ou seja, houve um aumento no número de vozes nos graus neutro e leve e diminuição de moderado e intenso. Contudo, após a técnica vocal, a maior parte das vozes ainda estava fora da área de normalidade.

**Tabela 3:** Relação entre o grau geral do desvio vocal e a área do diagrama de desvio fonatório, nos momentos pré e pós técnica vocal (n: 30)

**Área**	**Dentro**		**Fora**		**p-valor**
Pré	N	%	N	%	**0,000** *
Grau neutro	8	26,7	5	16,7	
Grau leve	5	16,7	3	10,0	
Grau moderado	0	0,0	7	23,3	
Grau intenso	0	0,0	2	6,7	
Grau extremo	0	0,0	0	0,0	
Total	13	43,34	17	56,67	
**Pós**	**N**	**%**	**N**	**%**	
Grau neutro	10	33,3	4	13,3	
Grau leve	4	13,3	6	20,0	
Grau moderado	0	0,0	5	16,7	
Grau intenso	0	0,0	1	3,3	
Grau extremo	0	0,0	0	0,0	
Total	14	46,6	16	53,3	

* Valores significativos: p ≤ 0,05 - Teste Mantel-Haenszel

**Legenda:** n = tamanho da amostra; % = percentual da amostra

Na análise visual qualitativa, verificou-se o deslocamento do DDF no gráfico, para o quadrante inferior esquerdo, ou seja, para mais próximo do quadrante representativo de normalidade, em 70,58% dos casos, após a aplicação da técnica.

A fim de ilustrar o deslocamento de uma voz após a técnica vocal, foram selecionados os diagramas, pré e pós técnica, de um participante deste estudo (Figura 3). Observa-se que a amostra vocal está fora da área de normalidade, nos momentos pré e pós técnica vocal. Qualitativamente, é possível observar o deslocamento do DDF para a esquerda, no eixo horizontal, após a técnica vocal.

**Figura 3:** Distribuições de amostras vocais de um sujeito desta pesquisa, pré e pós técnica vocal

**Legenda:** a = amostra vocal pré técnica vocal; b = amostra vocal após técnica vocal

## DISCUSSÃO

O DDF é uma ferramenta alternativa para avaliação e monitoramento da intervenção de vozes com desvios extremos^(14-16)^. Nesse contexto, pode-se considerar sua aplicação na população com DP, pois as alterações da qualidade vocal, a diminuição da intensidade da voz e a diminuição na amplitude da articulação estão entre as principais limitações na comunicação desses indivíduos^(8)^. Nesse contexto, a presente pesquisa se propôs a investigar o desempenho do DDF e de suas medidas no monitoramento da qualidade vocal em indivíduos com DP, após a técnica vocal de empuxo associada aos sons plosivos.

Para tanto, visando embasar os resultados do desempenho do DDF, foram analisados também os resultados de outros parâmetros acústicos isolados e da avaliação perceptivo-auditiva, antes e após a técnica vocal aplicada. Encontrou-se que houve diminuição na média dos valores de *shimmer*, sendo esse o único parâmetro acústico que apresentou diferença estatisticamente significante entre as médias pré e pós técnica vocal.

Embora esses resultados estejam acima dos valores de corte (6,5%) para a população vocalmente saudável, pode-se inferir que a melhora do valor do *shimmer*, após a execução do exercício, esteja relacionada à possível melhora da resistência glótica, considerando-se que esse parâmetro é sensível à diminuição da resistência glótica, além de ser correlacionada à presença de rugosidade na voz^(22)^. De maneira geral, a melhora da resistência glótica está associada ao aumento da intensidade vocal e é um dos principais objetivos terapêuticos na DP.

O *jitter* é uma medida fortemente correlacionada ao grau do desvio vocal^(23)^. No presente estudo, não houve diferença estatisticamente significante entre as médias de *jitter* pré e pós exercício vocal, mas vale destacar que, ainda assim, esse parâmetro apresentou valores de normalidade após a técnica vocal, o que pode ser um preditor de que houve melhora global da voz. Esse resultado concorda com a APA, visto que, após a técnica vocal, houve um aumento do número de vozes nos graus neutro e leve e diminuição nos graus moderado e intenso.

Ressalte-se que, além da maior parte ser composta por idosos, nos quais já se espera piora dos parâmetros acústicos devido às transformações morfológicas que ocorrem nas pregas vocais^(24)^, os participantes do presente estudo ainda têm DP, cujos parâmetros acústicos de *jitter* e *shimmer* aumentam significativamente^(25)^.

A análise da distribuição dos sinais vocais foi realizada de acordo com: área, quadrante, densidade e forma. Quando comparados os momentos pré e pós técnica vocal, não houve diferença significante na distribuição das amostras vocais, após o exercício. Entretanto, observa-se que muitas vozes já se encontravam dentro da área de normalidade, e que isso pode ter influenciado em termos de proporção.

Além disso, deve-se destacar que, nos casos de doenças neurodegenerativas, o objetivo da atuação fonoaudiológica é promover alternativas para comunicação, tendo em vista estratégias que propiciem melhora dos sintomas, na medida do possível^(26)^. Sendo assim, quando se trata de indivíduos com DP, o tratamento fonoaudiológico pode possibilitar melhora na voz e minimizar o impacto da doença^(27)^, mas não necessariamente trazer a voz para um padrão de normalidade.

Um dos princípios da terapia fonoaudiológica na DP é promover fechamento glótico mais eficiente a fim de melhorar a característica hipofônica da emissão. Neste estudo, observou-se o desempenho do DDF na análise do efeito de uma técnica que favorece a coaptação das pregas vocais (empuxo com sons plosivos)^(9)^.

Diferentemente de outro estudo que analisou o DDF de distintos tipos de disfonias pré e pós-tratamento vocal^(16)^, o presente estudo não apresentou diferença significante quanto à forma, densidade e localização nos quadrantes, entre os momentos pré e pós-tratamento. No entanto, embora não tenha apresentado tais diferenças na distribuição das amostras, na análise qualitativa, considerando-se as proporções, observou-se tendência de deslocamento do DDF para a esquerda, no eixo horizontal, após a aplicação da técnica vocal.

Esse resultado demonstra um bom desempenho do DDF na detecção de melhora vocal, após o exercício, visto que tal deslocamento posicionou o diagrama na região mais próxima da área de normalidade, concordando com os achados acústicos de melhora dos valores de *shimmer* e melhora global na voz, nos resultados da APA.

Ademais, sabe-se que vozes tensas tendem a se localizar no quadrante inferior esquerdo^(13)^. Então, vê-se que o exercício vocal realizado promoveu um aumento da eficiência fonatória, ou seja, diminuição da hipofonia. Dessa forma, pode-se indicar o uso do DDF para monitorar a condição de hipofunção vocal presente na Doença de Parkinson.

Antes da técnica vocal, a maioria das vozes estavam no quadrante inferior esquerdo, havendo, ainda, um aumento desse quantitativo após exercício vocal. É importante destacar que grande parte das vozes analisadas se encontraram distribuídas em mais de um quadrante; a maior parte estava nos quadrantes inferior esquerdo e inferior direito, sendo esse último característico de vozes rugosas^(13)^. Tais achados corroboram a literatura que indica que a rouquidão é uma das principais alterações vocais presentes na DP^(8,9)^. Essa característica pode estar relacionada à instabilidade vibratória das pregas vocais observada nessa doença.

Um estudo^(15)^ sobre o desempenho do DDF na avaliação de vozes sintetizadas rugosas, também concluiu que vozes com maior grau de rugosidade localizam-se no quadrante inferior direito. Além disso, foi observado que a área e quadrante do DDF são capazes de discriminar vozes com rugosidade de vozes saudáveis. Entretanto, no presente estudo, parte das vozes neutras, nos momentos pré e pós técnica, estavam fora da área de normalidade do DDF.

Ainda sobre a relação entre o grau geral do desvio vocal e a área do DDF, nota-se que, antes da técnica vocal, a maior parte das vozes já estava nos graus neutro e leve. Não houve nenhuma voz no grau extremo. Após o exercício vocal, houve um aumento no número de vozes nos graus neutro e leve, e diminuição de moderado e intenso. Assim como ocorreu antes da técnica, nenhuma voz apresentou desvio vocal extremo.

No caso ilustrado nos resultados, observa-se que, após a técnica vocal, houve um deslocamento da voz para a esquerda, no eixo horizontal, mas não entrou para área de normalidade proposta. Autores originais do diagrama de rouquidão analisaram a distribuição de vozes de indivíduos com diferentes patologias vocais^(14)^. Em uma voz altamente alterada, após tratamento cirúrgico para câncer de laringe, houve melhora da voz após a reabilitação vocal, o que provocou um deslocamento significativo da voz dentro do DDF, porém, depois de três meses, ainda não havia entrado na área de normalidade estabelecida.

Isso concorda com os resultados da presente pesquisa, pois, ainda que a maioria das vozes de grau neutro, tanto no momento pré quanto no pós-técnica vocal, estivessem dentro da área de normalidade proposta pelo próprio DDF, algumas estavam fora. Dessa forma, deve-se considerar que se tratam de vozes com alterações decorrentes de uma doença neurodegenerativa, em que a técnica vocal não irá trazer, necessariamente, para um padrão de normalidade, mas promover uma melhor eficiência vocal, objetivando minimizar o impacto da doença na voz, como já discutido anteriormente.

Por fim, vê-se que, em termos de limitações deste estudo há que se considerar o tamanho da amostra (n=30) que, a despeito de ser próximo ao de outro estudo que visou a analisar o DDF em vozes disfônicas em geral (n=34)^(16)^ foi bastante inferior à amostra da pesquisa com vozes sintetizadas (n=871)^(15)^. No entanto, por ser uma população específica, com o mesmo tipo de doença de base, ainda que em diferentes graus, e considerando-se as limitações impostas pela própria doença para a locomoção desses pacientes até o local de atendimento, pode-se considerar que o número de sujeitos foi oportuno. No entanto, sugere-se a continuidade do estudo em uma amostra maior e em diferentes tipos de disartrofonias.

Ressalta-se, também que, como não houve diferença na distribuição dos sinais vocais dos pacientes pré e pós intervenção em relação aos parâmetros de descrição, nesses casos, sugere-se que o fonoaudiólogo realize análise qualitativa visual por meio de observação do deslocamento da voz dentro dos quadrantes, no monitoramento terapêutico dos pacientes, por meio do DDF. É válido reforçar que o DDF facilita a análise combinada dos resultados acústicos vocais e ilustra prováveis mecanismos fonatórios utilizados pelo paciente, durante a emissão da voz falada^(16)^ e a análise qualitativa visual sugerida no presente estudo pode ser útil nesse raciocínio clínico, no acompanhamento desses pacientes.

Além disso, destaca-se que o profissional pode também utilizar o DDF como uma ferramenta auxiliar no processo terapêutico, especialmente no acompanhamento da evolução dos casos, utilizando-o como uma forma de *feedback* visual para o paciente, que pode acompanhar o deslocamento do diagrama, como um resultado da melhora de sua voz, durante o tratamento.

## CONCLUSÃO

Não houve diferença na distribuição dos sinais vocais dos pacientes pré e pós intervenção em relação aos parâmetros de descrição (área, forma, densidade e quadrante). Entretanto, em uma análise qualitativa visual, observou-se o deslocamento do sinal vocal para esquerda no eixo horizontal, em direção à diminuição da irregularidade.
